# LncRNA WEE2-AS1 knockdown inhibits the proliferation, migration and invasion of glioma cells via regulating miR-29b-2-5p/TPM3 axis

**DOI:** 10.32604/or.2022.03536

**Published:** 2022-07-13

**Authors:** ZHEN JIA, ZHENGTING QIAN, YONG TANG, XIANG LI, YAN SHI, HENG XIN, YOUWU FAN, HEMING WU

**Affiliations:** 1 Nanjing Medical University, Nanjing, 210000, China; 2 Department of Neurosurgery, Nanjing First Hospital, Nanjing Medical University, Nanjing, 210006, China

**Keywords:** LncRNA WEE2-AS1, Glioma, miR-29b-25p, TPM3, Proliferation

## Abstract

Glioma is a general malignant tumor with a dismal prognosis. Long noncoding RNAs (lncRNAs) have been implicated in the initiation and processes of tumors. An investigation of the GEPIA database revealed that long noncoding RNA WEE2 antisense RNA 1 (WEE2-AS1) is upregulated in glioma tissues compared to normal brain tissues, and validation with quantitative real-time polymerase chain reaction (qRT–PCR) revealed that WEE2-AS1 expression was consistent with the database prediction. Fluorescence *in situ* hybridization (FISH) assays revealed that WEE2-AS1 was localized primarily in the cytoplasm. Clone formation experiment and EDU assay were used to detect cell proliferation ability, and Transwell assay was used to detect cell migration and invasion ability, Western-blot assay and immunofluorescence were used to determine TPM3 protein level. Functional experiments revealed that the downregulation of WEE2-AS1 impeded cell proliferation, migration, and invasion in glioma cell lines. Furthermore, downregulation of WEE2-AS1 suppressed tumor growth *in vivo*. Bioinformatics predictions and integrated experiments indicated that WEE2-AS1 promoted tropomyosin 3 (TPM3) expression by sponging miR-29b-2-5p. A dual-luciferase reporter assay was conducted to uncover the binding of WEE2-AS1 and miR-29b-2-5p and that of miR-29b-2-5p and TPM3. Additionally, a series of rescue assays showed that WEE2-AS1 promotes proliferation, migration, and invasion by targeting miR-29b-2-5p to regulate TPM3 expression. Ultimately, the results of this study indicate that WEE2-AS1 plays an oncogenic role in glioma and may promote further investigations of the diagnostic and prognostic value of WEE2-AS1 in glioma.

## Introduction

Glioma accounts for almost 81% of malignant brain tumors, is recognized as one of the most common primary intracranial malignancies and is characterized by high mortality and morbidity [1]. Glioma is classified by the World Health Organization (WHO) into four categories (I–IV). Grade IV glioma (GBM) has the worst prognosis, with an average overall survival time of less than 2 years [2]. However, tremendous progress has been made in treatment strategies. Complete tumor resection, tumor treating fields (TTFields), radiotherapy and chemotherapy have been introduced into the treatment of glioma; however, glioma, particularly glioblastoma, is still associated with a rather high mortality and recurrence rate [3–6]. A thorough knowledge of the molecular process of glioma will assist in exploring new molecular biomarkers and potential molecular therapeutic targets.

Over the last decade, much research has been conducted on the nonprotein coding region of the human genome, which was formerly referred to as junk DNA [7]. Studies have demonstrated that most sequences transcribed from DNA in the genome have no protein-coding capability; these sequences are regarded as noncoding RNAs (ncRNAs) and include housekeeping and regulatory RNAs, the latter of which are divided into two groups based on length. ncRNAs of fewer than 200 nucleotides (nt) in length, such as microRNAs (miRNAs), piwi-interacting RNAs (piRNAs), and small interfering RNAs (siRNAs), are classified as small noncoding RNAs (sncRNAs), whereas ncRNAs with a length greater than 200 nucleotides (nt) are classified as long noncoding RNAs (lncRNAs) [8]. Accumulating evidence has revealed that the abnormal expression of lncRNAs is involved in a variety of physiological and pathological processes that contribute to the development of cancers, including glioma [9]. Moreover, lncRNAs have been proven to serve as competitive endogenous RNAs (ceRNAs) [10]. For example, lncRNA SNHG9 contributes to the progression of glioma through the miR-326/SOX9 axis [11]. GEPIA analysis revealed that WEE2-AS1 was substantially expressed in glioma tissues. Moreover, our subsequent bioinformatics analysis showed a candidate WEE2-AS1/miR-29b-2-5p/TPM3 axis in glioma. The miR-29 family consists of three members: miR-29a, miR-29b, and miR-29c, while miR-29b consists of miR-29b1 and miR-29b2 [12,13], this family is not only involved in many biological processes, including proliferation, survival, apoptosis and angiogenesis, but is also associated with tumor diseases [12]. MiR-29b-2-5p acts as a cancer suppressor gene for pancreatic ductal adenocarcinoma and promotes p53 expression by targeting CCL-B [14]. Tropomyosins (TPMs) are a series of thin filament proteins found in skeletal muscle and smooth muscle, as well as nonmuscle tissues [15]. TPM3 is located in region 1q21.3 and contains 1 exon, a member of the TPM family, which has the function of stabilizing cytoskeletal microfilaments [16,17]. TPM3 has been linked to malignant disorders in recent investigations. For example, platelet TPM3 mRNA is transferred to cancer cells through microcapsules and facilitates the migration of breast cancer cells [18]. TPM3 regulates the production of MMP2/9, which boosts the proliferation, migration, and metastatic potential of esophageal cancer cells as well as the epithelial-mesenchymal transition (EMT) [16]. However, the precise chemical mechanism by which WEE2-AS1 acts in glioma deserves more investigation.

In our study, based on data from the GEPIA website, our research indicated that glioma tumors overexpressed WEE2-AS1 in comparison to normal tissues. The expression of WEE2-AS1 was calculated by qRT–PCR in glioma cells and tissues. Next, a range of gain-of-function and loss-of-function tests were used to confirm the function of WEE2-AS1 *in vitro* and *in vivo*. Additionally, we explored the potential molecular mechanism by which WEE2-AS1 acts as an oncogene in glioma by regulating miR-29b-2-5p/TPM3. In general, the study conducted found that WEE2-AS1 may be a valuable biomarker for early detection and clinical therapy in glioma.

## Materials and Methods

### Clinical samples

Thirty glioma tissues before antitumor treatment were acquired from Nanjing Hospital Attached to Nanjing Medical University during the period of 2018–2021. Normal brain tissues were resected from accidental trauma patients. All samples were swiftly frozen with liquid nitrogen and maintained at a temperature of −80°C after surgical resection. All acquired tissues were histologically and pathologically diagnosed by two seasoned pathologists. The investigation was approved by the Ethics Committee of the Nanjing Hospital Affiliated to Nanjing Medical University. All participants were informed and completed an informed permission form.

### Cell lines

Procell (Wuhan, China) glioma cell lines (U87, T98, U251, and U118) were obtained and cultured in Dulbecco’s modified Eagle’s medium (DMEM) supplemented with 10% fetal bovine serum (FBS, Gibco Company, Grand Island, NY, USA). The ScienCell Research Laboratories provided normal human astrocytes (NHAs), which were cultivated in astrocyte medium (AM, ScienCell, Carlsbad, CA, USA, Cat. No. 1801). All six cell lines were cultured at 37°C in a humidified incubator containing 5% CO_2_.

### Cell transfections

GenePharma (Shanghai, China) generated short hairpin RNA WEE2-AS1 (sh-WEE2-AS1), short hairpin RNA TPM3 (sh-TPM3), a WEE2-AS1 expression plasmid (WEE2-AS1), a TPM3 expression plasmid (TPM3), hsa-miR-29b-2-5p mimics, a hsa-miR-29b-2-5p inhibitor, and a negative control inhibitor, which are displayed in Table 1. As part of the transfection process, Lipo3000 (Invitrogen, Waltham, MA, USA) was used in line with the manufacturer’s instructions. The EGFP-Luciferase-SV40 lentivirus vectors were constructed by Genechem Biotechnology (Shanghai, China), and the transfection steps were conducted in line with the instructions provided by the manufacturer.

**Table 1:** The interfering nucleotides used in this study

Plasmid sequences	Sequence	Sequence
	Sense(5’-3’)	Antisense(5’-3’)
sh-WEE2-AS1-1	GCAACTGAAACTGTCATAT	
sh-WEE2-AS1-2	GCGAAGTTCCCAGGAGTAAGA	
hsa-miR-29b-2-5p mimics	CUGGUUUCACAUGGUGGCUUAG	AAGCCACCAUGUGAAACCAGUU
hsa-miR-29b-2-5p inhibitor	CUAAGCCACCAUGUGAAACCAG	
sh-TPM3	GGAACTCCAGGAAATCCAACT	
inhibitor NC	CAGUACUUUUGUGUAGUACAA	
sh-NC	GTTCTCCGAACGTGTCACGT	
NC	UUCUCCGAACGUGUCACGUTT	ACGUGACACGUUCGGAGAATT

### Colony formation assay

Transfected or nontransfected cells were collected (500 cells/well) and plated in 6-well plates. The cells were rinsed with phosphate buffered saline (PBS; Gibco Company, Grand Island, NY, USA) after 14 days of growth at 37°C, immobilized with 4% formaldehyde (Servicebio, Wuhan, China) for 20 min and stained with 0.5% crystal violet for 10 min (Beyotime, Shanghai, China). Colonies with a minimum of 50 cells were photographed and manually counted.

### Quantitative reverse transcription polymerase chain reaction

TRIzol reagent (Vazyme, Nanjing, China) was utilized to extract total RNA from cells and tissues following the manufacturer’s instructions. Complementary deoxyribonucleic acid (cDNA) was synthesized using HiScript III RT SuperMix for qPCR (+gDNA wiper) and a miRNA 1st Strand cDNA Synthesis Kit (by stem–loop) (Vazyme). Following the instructions for ChamQ Universal SYBR qPCR Master Mix (Vazyme), the expression level was measured using qRT–PCR. The relative gene expression was calculated by the 2^−ΔΔCt^ technique and standardized to GAPDH or U6. Table 2 contains the sequences of primers.

**Table 2:** Chemical compositions of base alloys for preparing multicomponent diffusion multiples (wt.%)

Primer	Sequence	Sequence
	Forward	Reverse
LncRNA WEE2-AS1	CTCTGTTCGGCATCAGGTTGGAT	GCTGGGTTGGAGTTGGCTTGA
hsa-miR-29b-2-5p	ATTGGCTGGTTTCACATGGTG	GTGCAGGGTCCGAGGT
TPM3	GATCCAGCTGGTTGAAGAAGAG	ACCTTCATACCTCTCTCACTCT
GAPDH	CATGAGAAGTATGACAACAGCCT	AGTCCTTCCACGATACCAAAGT
U6	CAGCACATATACTAAAATTGGAACG	ACGAATTTGCGTGTCATCC

### 5-Ethynyl-2’-deoxyuridine cell proliferation assay

The Cell-Light KFluor555 EdU kit (KeyGEN, Jiangsu, China) was used to monitor glioma cell growth. For the EdU experiment, transfected cells (4 × 10^4^) were plated in 24-well plates. To determine cell proliferation, U87 and U251 cells were treated for 2 h with EdU solution (10 µM). After fixation with 4% formaldehyde (Servicebio), 0.5% Triton X-100 was applied to infiltrate the cells. A Click-iT reaction mixture was utilized to stain the cells, and counterstaining was performed using DAPI (Beyotime). Five random fields were photographed via a fluorescence microscope (Carl Zeiss Microscopy, Oberkochen, Germany).

### Fluorescence in situ hybridization assay

FISH assays were performed to identify the subcellular distribution of WEE2-AS1 following the standard protocol of GenePharma lncRNA FISH Probe Mix (Red). The sequence of FISH probe was displayed in Table 3. In a 24-well plate, glioma cells were sown at a density of 2 × 10^4^ cells per well. After treatment with 4% paraformaldehyde (Servicebio), the cells were rinsed with PBS, permeabilized, and treated with prehybridization solution. Next, the cell samples were hybridized overnight at 37°C with the WEE2-AS1 probe without light exposure. Following elution, DAPI was used to label nuclei for fluorescence microscopy (Cari Zeiss Microscopy).

**Table 3:** The FISH probes used in this study

RNA FISH Probe KIT B	Sequence
LncWEE2-AS1 homo probe	TGACGCAATCC+TCAGACCT+TACCCAC
LncWEE2-AS1 homo probe1	GGACCT+TAAAGACACTGCC+TAGCCCTCT
LncWEE2-AS1 homo probe2	CACT+TTGAAATGAGAAA+TGTGATGGGCAA

### Western blot analysis

RIPA tissue cell lysis buffer (YIFEIXUE BIO TECH, Nanjing, China) was utilized to obtain the total protein of cells and glioma specimens. The content was determined using a BCA quantification kit (YIFEIXUE BIO TECH). Primary antibodies against TPM3 (1:1000, ProteinTech Group, Inc., Wuhan, China) and β-actin (1:3,000, ProteinTech Group) was used in this study. The steps were the same as those described in previous study [19].

### Transwell migration and invasion assay

A migration assay was conducted in a 24-well Transwell chamber (Millipore Corporation, Billerica, MA, USA), while an invasion assay was carried out utilizing a Transwell chamber precoated with Matrigel solution (BD Biosciences, San Jose, CA, USA). In 300 μl of serum-free media, 2 × 10^4^ transfected U87 and U251 cells were introduced to the top chamber. The bottom compartments were filled to capacity with 500 µl DMEM containing 10% FBS. Cotton swabs were employed to clean cells from the top chambers after 48 h of incubation, while cells breaching the membranes were preserved with methanol and subsequently stained with 0.1% crystal violet. Finally, photos of cells were taken using a NIKON T1 microscope (NIKON, Tokyo, Japan).

### Dual-luciferase reporter gene assay

The wild-type (WT) WEE2-AS1 or TPM3 sequences containing the target site of miR-29b-2-5p were amplified and introduced into the pmiRGLO luciferase reporter plasmid to establish WEE2-AS1-WT and TPM3-WT plasmids. Mutant type (MUT) vectors were constructed. The sequences of all constructed vectors were synthesized by GeneChem and shown in Table 4. Well-constructed vectors were cotransfected with miR-29b-2-5p mimic or NC into U87 and U251 cells using Lipofectamine 3000 (Invitrogen, USA) following the manufacturer's recommended protocols. The cells were extracted after 48 hours of incubation. The relative luciferase activity in cells was evaluated using a dual-luciferase-reporter-gene system (Promega, Madison, WI, USA). As an internal control, Renilla luciferase was used.

**Table 4:** The Dual-luciferase plasmid sequences used in this study

Dual-luciferase plasmid	Sequence
LncWEE2-AS1-WT	CCCCCACTACAACAAATTATGCAGTCGAGTTTTCCACATTTGGGAATATCACAGGGGTCAGCACATCCGGAGTGCAATCGATGAGCCCCACGCTGGGAAAAACCACCTTCGTGATCATGGTATCTTCCCTGCCAGGGAAGGCCGGAGAACTGTATTTCTGGCCAGAGCTGCTGCAGAAGGTCTCTGTTCGGCATCAGGTTGGATCATTAATATTGACCAGAGCCAATGAGGTCATCTCATCCTTGAGGGGAGCAGGTGTGAGCTTCAAATGCTTAGGGCCTCTGGAAGGAAGCTTCCCTGTTGGAGAAATCACCAACCGGCTCAGCATGGTCTGAATGAGGATGACAAAAGGAGTTGCTGCACAGGGGGAAAGCGAAGTTCCCAGGAGTAAGATGTGCAAGAAGCGGGCAGCGGAAAATCTATCTATTCAAATTTGCAAGACGTGGAAACTTTTATTCACCTTCCTGGTTAGACTGTTCCTTTGTCAAAGTAAAGCTAAGGCCATGGAAAATGAGCTATTTGAGAGTTTGTCGCTCCAAGGAAGAACATGCTGGCCTAATTGTCCCCCAAGTCAGGAGAGAGAATTGTAAAAAACACTTAAACAGGCCTAAGTAAAGG
LncWEE2-AS1	CCCCCACTACAACAAATTATGCAGTCGAGTTTTCCACATTTGGGAATATCACAGGGGTCAGCACATCCGGAGTGCAATCGATGAGCCCCACGCTGGGAAACCAACCCTTCGTGATCATGGTATCTTCCCTGCCAGGGAAGGCCGGAGAACTGTATTTCTGGCCAGAGCTGCTGCAGAAGGTCTCTGTTCGGCATCAGGTTGGATCATTAATATTGACCAGAGCCAATGAGGTCATCTCATCCTTGAGGGGAGCAGGTGTGAGCTTCAAATGCTTAGGGCCTCTGGAAGGAAGCTTCCCTGTTGGATCCCGACCCAACCGGCTCAGCATGGTCTGAATGAGGATGACAAAAGGAGTTGCTGCACAGGGGGAAAGCGAAGTTCCCAGGAGTAAGATGTGCAAGAAGCGGGCAGCGGAAAATCTATCTATTCAAATTTGCAAGACGTGGAAACTTTTATTCACCTTCCTGGTTAGACTGTTCCTTTGTCAAAGTAAAGCTAAGGCCATGGAAAATTCTAGCTTTGAGAGTTTGTCGCTCCAAGGAAGAACATGCTGGCCTAATTGTCCCCCAAGTCAGGAGAGAGAATTGTAAAAAACACTTAAACAGGCCTAAGTAAAGG
TPM3-WT	AGACCAGCCTGGCAATAAAGTGAGAACCTGTCTCTACAAAATATAAAAACTTAGCCAAACATGGTGGTGCGTGCCTGCAGTCCTATCTACTTGGAGGGCCGAAGCCAGAGGATCCTTTGAGCCCAGGAGTCTGAGGCTGCAGCAAGCTGTGATCACACTGCTGCACTCCATCCTGGGTGGCAGAATGAGACCCCCCCCAAAAAAAGAGAGGAAATAAGAGGGAGATTGGGAGAAAAGTAAGGTGTTATAGCTCTGATCTGTGGTATCTCCTGTTTTCCTTGCGGTATGACTAGTGTGAGCCAAGGATAGAGACCAACAAACTGGGGTACCAAAGTGGACAATGAAGTACTATGTAATTAGTGCTAATGCTAAATTCATTCCTTTGTTTAAGGCTTAATATCCTTGCAGAAGCCATCCATGGGGTTGTTTCTTAATTAGATATAGCTGAAAAGAACGCTAGACCAAATAGGTTCTCTGCCTTGCCTTTTCGTTTGTTTTGTTTTAGCTATTATCAGGGAACCAAAAACTTTAAGGAGCTAGTACTGGTCTTAATTTTTAATAACTAGAGATAGCAGAGTTAGAAACTAAGTTCAAAGTGAGAGAACAGCTGCATTTGTCTTTC
TPM3-MUT	AGACCAGCCTGGCAATAAAGTGAGAACCTGTCTCTACAAAATATAAAAACTTAGCCAAACATGGTGGTGCGTGCCTGCAGTCCTATCTACTTGGAGGGCCTCCTAACTAGGATCCTTTGAGCCCAGGAGTCTGAGGCTGCAGCAAGCTGTGATCACACTGCTGCACTCCATCCTGGGTGGCAGAATGAGACCCCCCCCAAAAAAAGAGAGGAAATAAGAGGGAGATTGGGAGAAAAGTAAGGTGTTATAGCTCTGATCTGTGGTATCTCCTGTTTTCCTTGCGGTATGACTAGTGTGAGCCAAGGATATCTCAACACAAACTGGGGTACCAAAGTGGACAATGAAGTACTATGTAATTAGTGCTAATGCTAAATTCATTCCTTTGTTTAAGGCTTAATATCCTTGCAGAAGCCATCCATGGGGTTGTTTCTTAATTAGATATAGCTGAAAAGAACGCTAGACCAAATAGGTTCTCTGCCTTGCCTTTTCGTTTGTTTTGTTTTAGCTATTATCAGTTCCAACAAAACTTTAAGGAGCTAGTACTGGTCTTAATTTTTAATAACTAGAGATAGCAGAGTTAGAAACTAAGTTCAAAGTGAGAGAACAGCTGCATTTGTCTTTC

### Xenograft mouse models

The Ethics Committee of Nanjing First Hospital granted clearance for the following studies. The Animal Center of Nanjing First Hospital provided twelve specific pathogen-free male (SPF) BALB/c athymic nude mice, which were randomly split into two groups. The nude mice were housed in an SPF laminar air flow room at a suitable temperature (22°C–26°C) and humidity (55 ± 5%). The right frontal lobes of specific pathogen-free male BALB/c athymic nude mice were injected with 2 × 10^5^ U87-sh-NC or U87-sh-WEE2-AS1 cells expressing luciferase. Tannon 5200 imaging equipment (Shanghai, China) was used to determine *in vivo* imaging intensity every 10 days following implantation. Mouse survival rates were calculated after all experimental nude mice were sacrificed. Whole-brain tissues were extracted from nude mice for hematoxylin-eosin staining, qRT–PCR and western blot assays.

### Immunohistochemical staining

Clinical specimens were fixed in paraffin and subsequently sectioned into 4-μm sections, which were dewaxed and blocked using 5% normal goat serum after hydration, blocking, and antigen repair. Then, the paraffin-embedded tissues were treated with a primary antibody directed against TPM3 for 12 h at 4°C (1:500 Abcam, Waltham, MA, USA). After three PBS washes, the slices were treated with the secondary antibody (1:200 Abcam) for 1 h at 37°C. Finally, diaminobenzidine was used to stain the sections, followed by hematoxylin for counterstaining. A NIKON T1 microscope (NIKON) was used to acquire the experimental data.

### Differential expression analysis and survival analysis

The R programming language (3.6.3) was used to examine gene expression patterns in gliomas with a variety of clinicopathological characteristics. The overall survival of patients who were diagnosed with glioma was assessed using Kaplan–Meier survival analyses, which were separated into two groups based on WEE2-AS1 median expression levels: high expression and low expression. Using the R package “survival,” the log-rank test was used to assess the difference in patient survival.

### Immunofluorescence staining assay

Transfected cells (U87 and U251) were cultured at a density of 70% on glass coverslips overnight, immobilized with 4% paraformaldehyde (Servicebio) for 10 min, and then permeabilized with 0.5% Triton X-100 (Solarbio, Beijing, China) for 20 min. The cells were then blocked for 1 h at room temperature with 10% equine serum (Solarbio). The cells were then treated overnight at 4°C using TPM3 antibody (1:1000, Abcam), followed by 1 h at 37°C with goat anti-mouse IgG (1:200, ProteinTech Group). Then, the nuclei were counterstained with DAPI. Finally, Cari Zeiss Microscopy was used to capture the cells.

### Hematoxylin-eosin staining

The 3 μm-paraffin slices were dewaxed and rinsed with water before being treated with hematoxylin dye. After dehydrating the sections using an anhydrous ethanol gradient, they were colored with eosin and sealed with neutral glue. Finally, using a microscope, the pictures are gathered and examined.

### Bioinformatics analysis

The TCGA data portal (https://tcga-data.nci.nih.gov/TCGA/) was used to gather gene expression data and clinical data (698 cases, Workflow Type: HTSeqCounts). The publication criteria established by TCGA (https://cancergenome.nih.gov/publications/publishing-guidelines) were strictly followed in this work. The overall survival of glioma patients was assessed using Kaplan–Meier survival analyses. Patients were divided into high and low expression groups based on the median level of WEE2-AS1 expression. The log-rank test was performed to evaluate the difference in patient survival using the R package “survival.” DIANA TOOLS (https://dianalab.e-ce.uth.gr/tools), LNCediting, (http://bioinfo.life.hust.edu.cn/LNCediting/) and RNA 22 (https://cm.jefferson.edu/rna22/) were used to predict the target miRNAs of WEE2-AS1. The highly rated predicted targets of WEE2-AS1 were tested using qRT–PCR, and miR-29b-2-5p was found to be a miRNA of interest. The TargetScan (http://www.targetscan.org/vert_72/), miRWalk (http://mirwalk.umm.uni-heidelberg.de/), mirTarbase, (http://miRTarBase.cuhk.edu.cn/), and TargetMiner databases (https://www.isical.ac.in/~bioinfo_miu/targetminer20.htm) were used to confirm the target genes of miR-29b-2-5p. qRT–PCR was verified using the intersection of the anticipated target genes provided. MiR-29b-2-5p-binding sites were predicted in TPM3’s 3’-UTR.

### Statistical analysis

SPSS 23.0 (Armonk, NY, USA) statistical software was used to analyze all of the data. The average and standard deviation (SD) of three different experiments are presented. GraphPad Prism V9.00 (GraphPad Software, Inc., La Jolla, CA, USA) was used to produce the figures. To examine differences, Student’s *t* test or one-way ANOVA was utilized. Overall survival was calculated using the Kaplan–Meier method. The associations between WEE2-AS1 and miR-29b-2-5p expression, as well as miR-29b-2-5p and TPM3 expression in clinical samples, were investigated using Pearson correlation analysis. *p* < 0.05 was considered statistically significant.

## Results

### The expression of lncRNA WEE2-AS1 was dramatically increased in glioma tissues and cell lines

**Figure 1:**
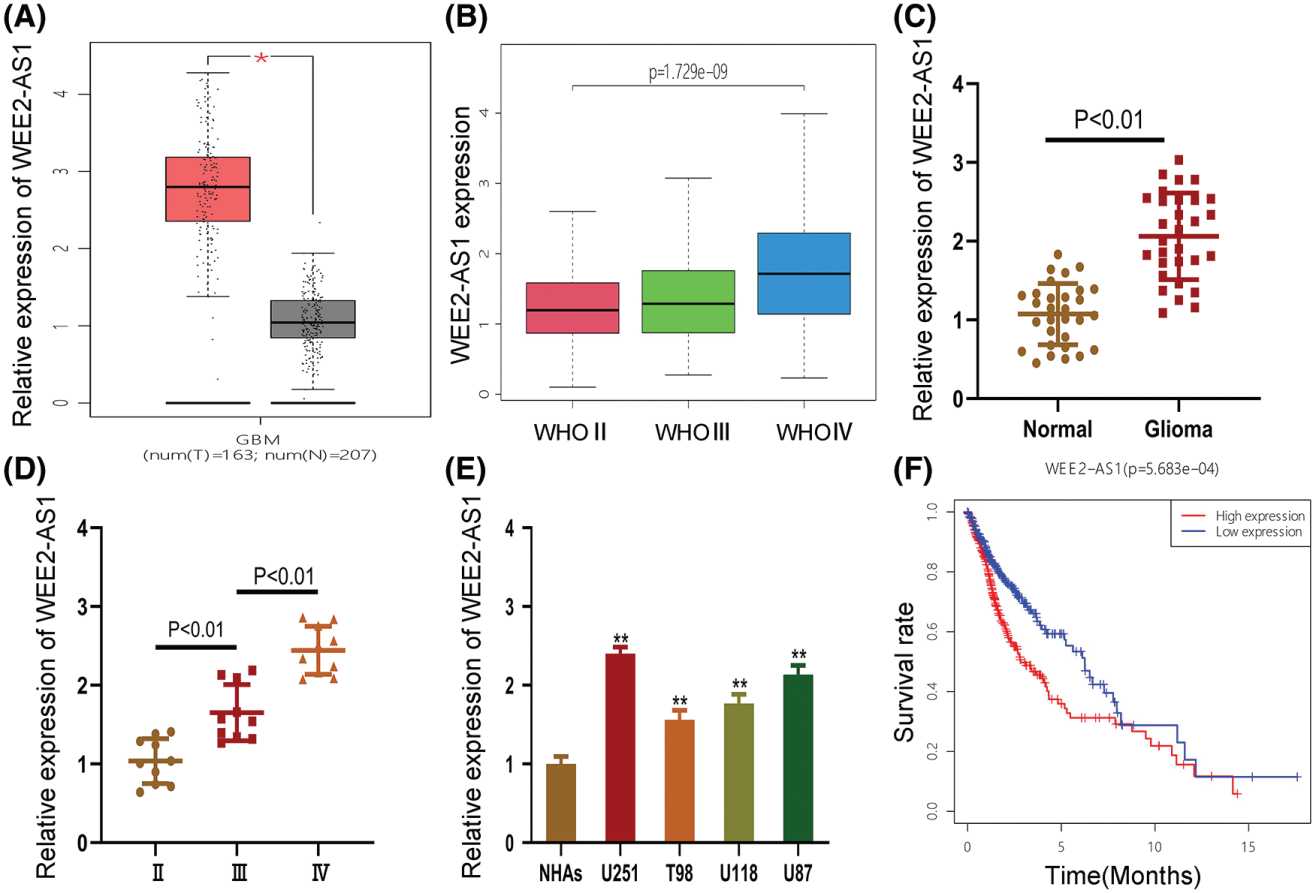
WEE2-AS1 expression is increased in glioma tissues and cell lines. (A) The relative expression of WEE2-AS1 was identified within the TCGA dataset. (B) The expression levels of WEE2-AS1 in different grades of glioma was evaluated using the TCGA database. (C) The expression levels of WEE2-AS1 in clinical specimens. (D) The differential expression of WEE2-AS1 in gliomas of different grades. (E) The relative expression of WEE2-AS1 in NHAs and glioma cell lines. (F) The survival package in the R software was used to examine the information survival rate of TCGA clinical samples. Comparison was analyzed by *t*-test for (C) and (E). Comparison was analyzed by one-way ANOVA for (D). Data are expressed as the mean ± SD *p < 0.05, **p < 0.01 from three independent experiments.

We discovered that WEE2-AS1 expression was higher in glioma tissue than in normal tissue using the online analytic platform GEPIA (gepia.cancer-pku.cn/) (Fig. 1A). To explore the differential expression of WEE2-AS1 in WHO grades II, III and IV gliomas, clinical data for 698 gliomas of different WHO types were gathered from the TCGA-GDC database and analyzed (Fig. 1B). To ascertain WEE2-AS1 expression in glioma, qRT–PCR was employed on 30 glioma and normal tissues. The results showed that WEE2-AS1 was drastically higher in glioma than in normal brain tissues and that the expression of WEE2-AS1 increased with WHO grade, which was similar to the predicted result based on TCGA database (Figs. 1C and 1D). Moreover, we detected WEE2-AS1 expression in glioma cells and normal human astrocytes (NHAs) and observed that WEE2-AS1 was drastically increased in glioma cells (especially U87 and U251) compared to NHAs (Fig. 1E). Thus, we chose the U87 and U251 cell lines for subsequent studies. Furthermore, clinical data downloaded from TCGA database were analyzed by R statistical software. On the basis of the median level of WEE2-AS1 expression, patients were split into two groups: those with high expression and those with low expression. The Log-Rank test was used to analyze variations in survival rates using the R software package “Survival”. The results revealed that glioma patients with higher expression levels of WEE2-AS1 had markedly worse overall survival than those with lower expression levels of WEE2-AS1 (Fig. 1F).

### LncRNA WEE2-AS1 accelerates cell proliferation, migration and invasion in glioma

**Figure 2:**
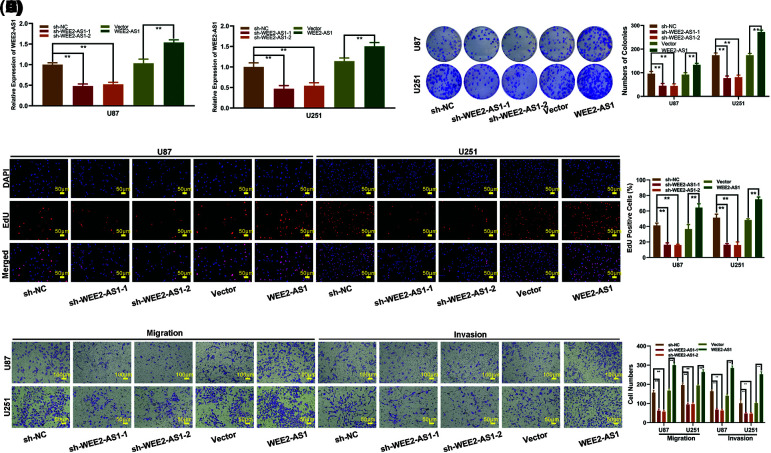
WEE2-AS1 stimulates the proliferation, migration, and invasion of glioma cells *in vitro*. (A, B) The relative expression of WEE2-AS1 was evaluated by qRT–PCR in U87 and U251 cells transfected with sh-WEE2-AS1 (including sh-WEE2-AS1-1 and sh-WEE2-AS1-2), WEE2-AS1 plasmid, or a negative control plasmid. (C, D) The proliferation of U87 and U251 cells transfected with sh-WEE2-AS1, WEE2-AS1 plasmid or the corresponding negative control, as measured by clone formation assays. (E, F) The proliferation of U87 and U251 cells transfected with sh-WEE2-AS1, the WEE2-AS1 plasmid, or the corresponding negative control was determined using EdU assays. (G, H) Transwell assays were used to determine the migration and invasion of U87 and U251 cells transfected with sh-WEE2-AS1, the WEE2-AS1 plasmid, or the corresponding negative control. Comparison was analyzed by *t*-test for (A), (B), (D), (F) and (H). Data are expressed as the mean ± SD, **p < 0.01 from three independent experiments.

To establish the probable role of WEE2-AS1 in glioma, sh-WEE2-AS1, a WEE2-AS1 expression plasmid and a negative control were transfected into U87 and U251 cells, and qRT–PCR was applied to validate the transfection effectiveness (Figs. 2A and 2B). EdU tests and colony-formation experiments were adopted to evaluate the ability of glioma cell lines to proliferate (U87 and U251). The colony-formation assays (Figs. 2C and 2D) and the EdU assay (Figs. 2E and 2F) results revealed that U87 and U251 cell proliferation was restrained after the downregulation of WEE2-AS1, whereas cell proliferation was promoted under the upregulation of WEE2-AS1. To assess cell migration and invasion capacity, a Transwell test was used. The number of migrated glioma cells dramatically decreased after WEE2-AS1 was downregulated, while the upregulation of WEE2-AS1 had the opposite effect (Figs. 2G and 2H). The findings demonstrate that WEE2-AS1 is oncogenic for glioma development.

### LncRNA WEE2-AS1 functions as a sponge of miR-29b-2-5p

**Figure 3:**
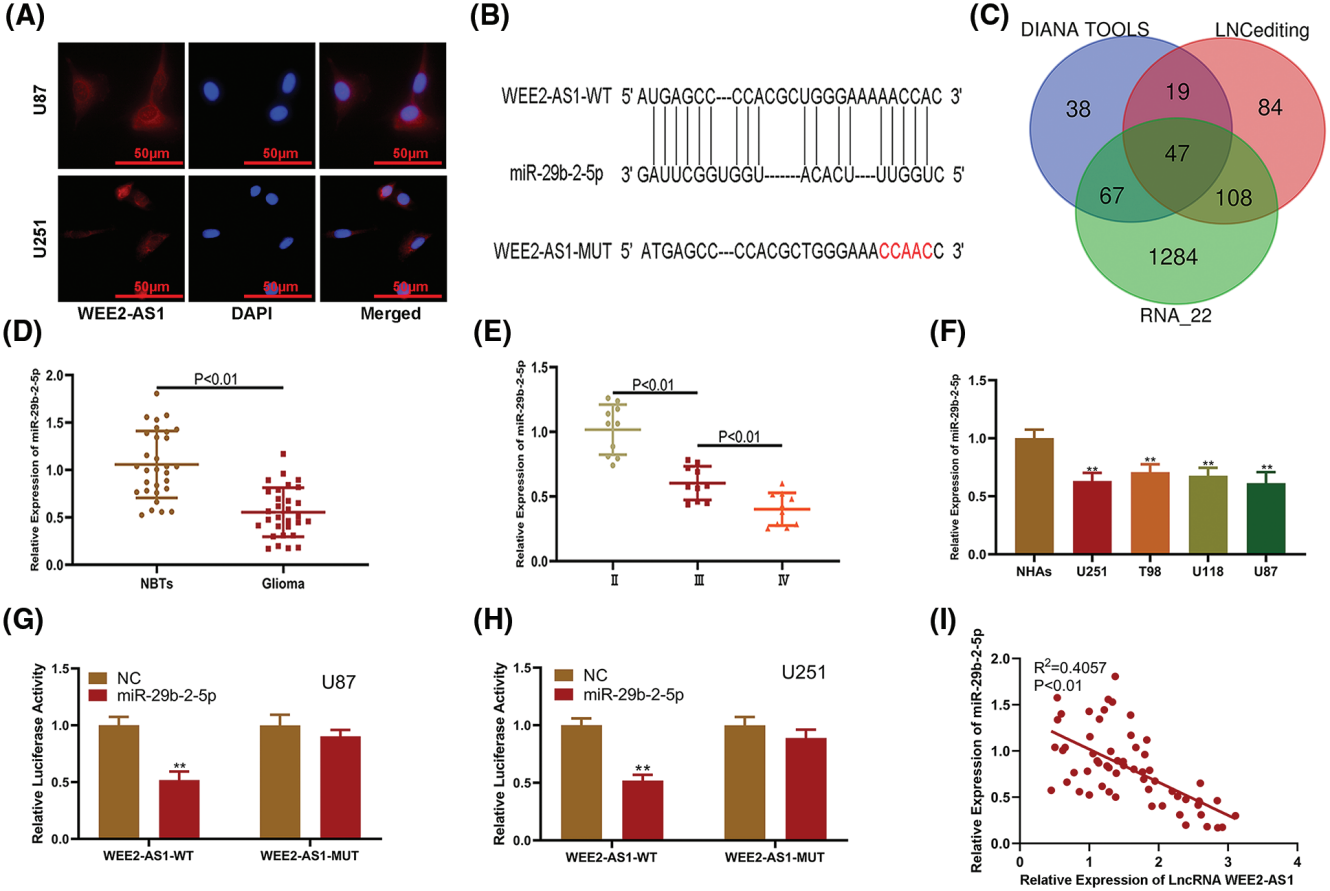
LncRNA WEE2-AS1 functions as a sponge of miR-29b-2-5p. (A) FISH tests were used to assess the location of WEE2-AS1 in U87 and U251 cells. (B) An online database suggested the probable miR-29b-2-5p binding sites on WEE2-AS1. (C) Venn diagram analysis of the intersection of differentially expressed databases (DIANA TOOLS, LNCediting and RNA 22). (D) The comparative expression of miR-29b-2-5p in tissue samples of normal brian and glioma. (E) The comparative expression of miR-29b-2-5p at different stages of glioma. (F) The level of miR-29b-2-5p expression in NHAs and glioma cell lines. (G) The miR-29b-2-5p inhibited the luciferase activity of WEE2-AS1-WT but not WEE2-AS1-MUT in U87 cells. (H) The miR-29b-2-5p inhibited the luciferase activity of WEE2-AS1-WT but not WEE2-AS1-MUT in U251 cells. (I) Pearson correlation between LncWEE2-AS1 and miR-29b-2-5p. Comparison was analyzed by *t*-test for (D), (F), (G) and (H). Comparison was analyzed by one-way ANOVA for (E). Pearson’s correlation analysis was employed to illustrate the relation for (I). Data are expressed as the mean ± SD, **p < 0.01 from three independent experiments.

FISH assays were carried out to verify the function of WEE2-AS1. WEE2-AS1 was chiefly concentrated in the cytoplasm of U87 and U251 cells, indicating that it may play a crucial role as a ceRNA by base pairing (Figs. 3A and 3B). Subsequently, to explore the potential target miRNAs of WEE2-AS1, we conducted a bioinformatics prediction assay with DIANA Tools, LNCediting and RNA_22. By analyzing the intersection among these databases, miR-29b-2-5p was identified as a miRNA of interest (Figs. 3C). Next, we quantified miR-29b-2-5p expression in clinical specimens and discovered that it was significantly higher in normal brain tissues than in glioma tissues (Figs. 3D and 3E). Moreover, qRT–PCR showed that the expression of miR-29b-2-5p was lower in glioma cell lines than in NHAs (Fig. 3F). Furthermore, luciferase reporter assays suggested that the miR-29b-2-5p mimics markedly decreased the luciferase activity of WEE2-AS1-WT but had almost no influence on WEE2-AS1- MUT (Figs. 3G and 3H). In addition, Pearson correlation analysis found an adverse connection between the expression of WEE2-AS1 and miR-29b-2-5p (Fig. 3I). Considering all the results together, we concluded that WEE2-AS1 might work as a ceRNA for miR-29b-2-5p in glioma cells.

### MiR-29b-2-5p targets TPM3 in glioma

**Figure 4:**
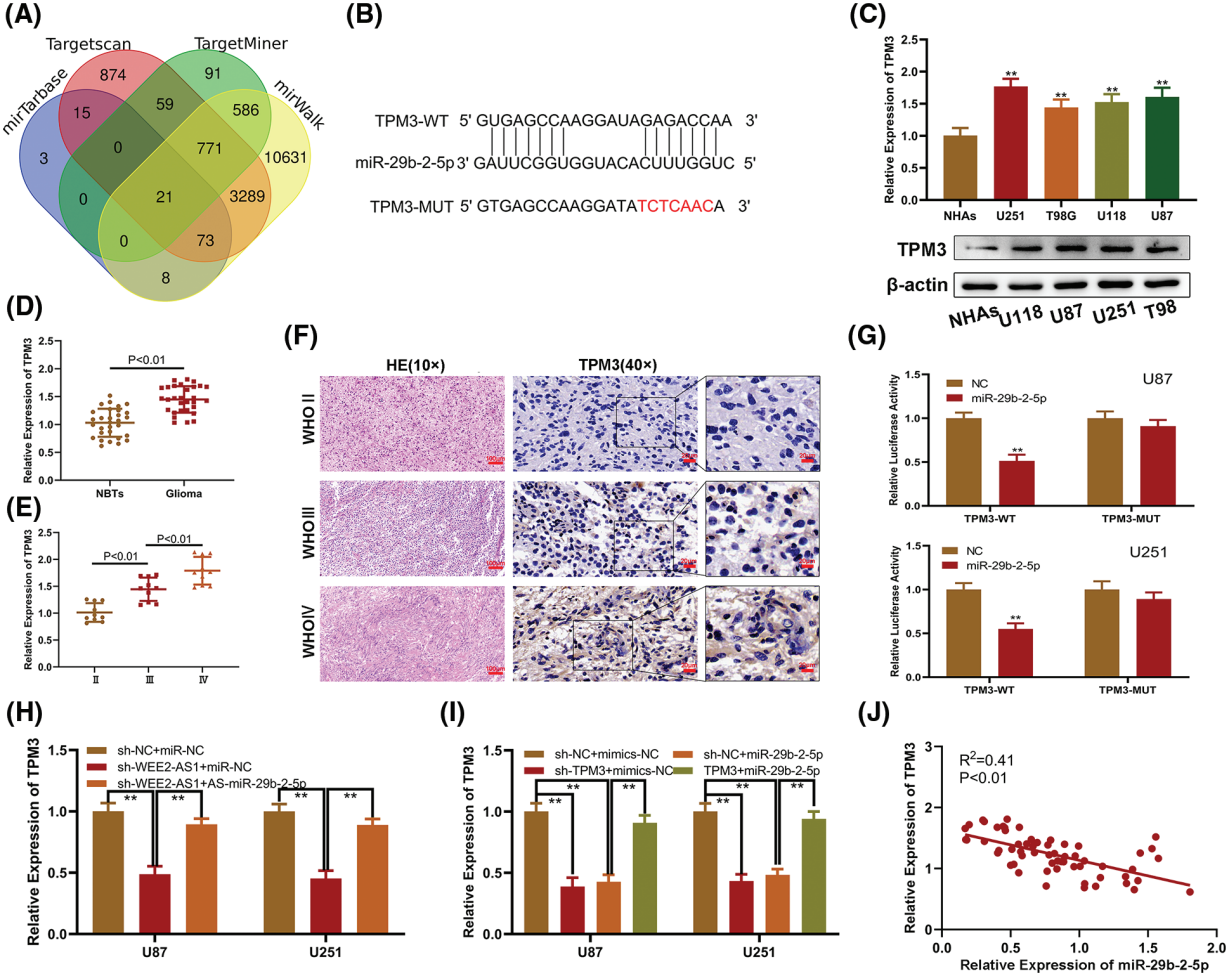
MiR-29b-2-5p targets TPM3 in glioma. (A) Venn diagram indicating the intersection of miR-29b-2-5p target genes predicted in public databases (mirTarbase, TargetScan, TargetMiner and mirWalk). (B) An online database suggested the probable miR-29b-2-5p binding sites on TPM3. (C) The level of TPM3 expression in NHAs and glioma cell lines measured by qRT-PCR and Western-blot assay. (D, E) The relative expression of TPM3 in clinical specimens and in different grades of glioma. (F) TPM3 expression in different glioma grades was revealed by immunohistochemistry. (G) MiR-29b-2-5p inhibited the luciferase activity of TPM3-WT but not TPM3-MUT in luciferase reporter test. (H). Relative expression of TPM3 in U87 and U251 cells transfected with sh-LncWEE2-AS1, miR-29b-2-5p inhibitor or the corresponding negative control, as measured by qRT-PCR. (I) Relative expression of TPM3 in U87 and U251 cells transfected with sh-TPM3, miR-29b-2-5p mimics, TPM3 expression plasmid or the corresponding negative control, as measured by qRT-PCR. (J) Pearson correlation between miR-29b-2-5p and TPM3. Comparison was analyzed by *t*-test for (C), (D), (G) one-way ANOVA for (E), (H) and (I). Pearson’s correlation analysis was employed to illustrate the relation for (J). Data are expressed as the mean ± SD, **p < 0.01 from three independent experiments.

A bioinformatics assay was employed to identify the downstream target gene of miR-29b-2-5p, and TPM3 was selected for validation due to its high score (Fig. 4A). TPM3 and miR-29b-2-5p were paired with complementary bases (Fig. 4B). We subsequently explored TPM3 expression in glioma cells relative to that in NHAs and discovered that TPM3 expression was elevated in the glioma cell lines measured by qRT–PCR and Western-blot assay (Fig. 4C). Furthermore, clinical specimens were subjected to qRT–PCR to validate TPM3 expression levels in glioma tissues *vs*. normal brain tissues (Fig. 4D and 4E). Furthermore, IHC analysis revealed a correlation between TPM3 expression and malignancy grade (Fig. 4F). Moreover, the luciferase reporter assay indicated that the miR-29b-2-5p mimics intensively suppressed the luciferase activity of TPM3-WT, while no effects were observed in TPM3-MUT, implying that miR-29b-2-5p targets TPM3 (Fig. 4G). qRT-PCR identified TPM3 downregulation in sh-WEE2-AS1 transfected glioma cell lines, and miR-29b-2-5p inhibitor reversed this impact (Fig. 4H). TPM3 was down-regulated when sh-TPM3 or miR-29b-2-5p mimics were transfected with glioma cell lines detected by qRT-PCR, which could be restored by a TPM3 overexpression plasmid (Fig. 4I). The expression of miR-29b-2-5p and TPM3 was negatively associated, according to Pearson correlation analysis (Fig. 4J). Based on our observations, we concluded that TPM3 might act as the target gene of miR-29b-2-5p.

### LncRNA WEE2-AS1 promotes the proliferation, migration, and invasion of glioma cells through miR-29b-2-5p sponging

**Figure 5:**
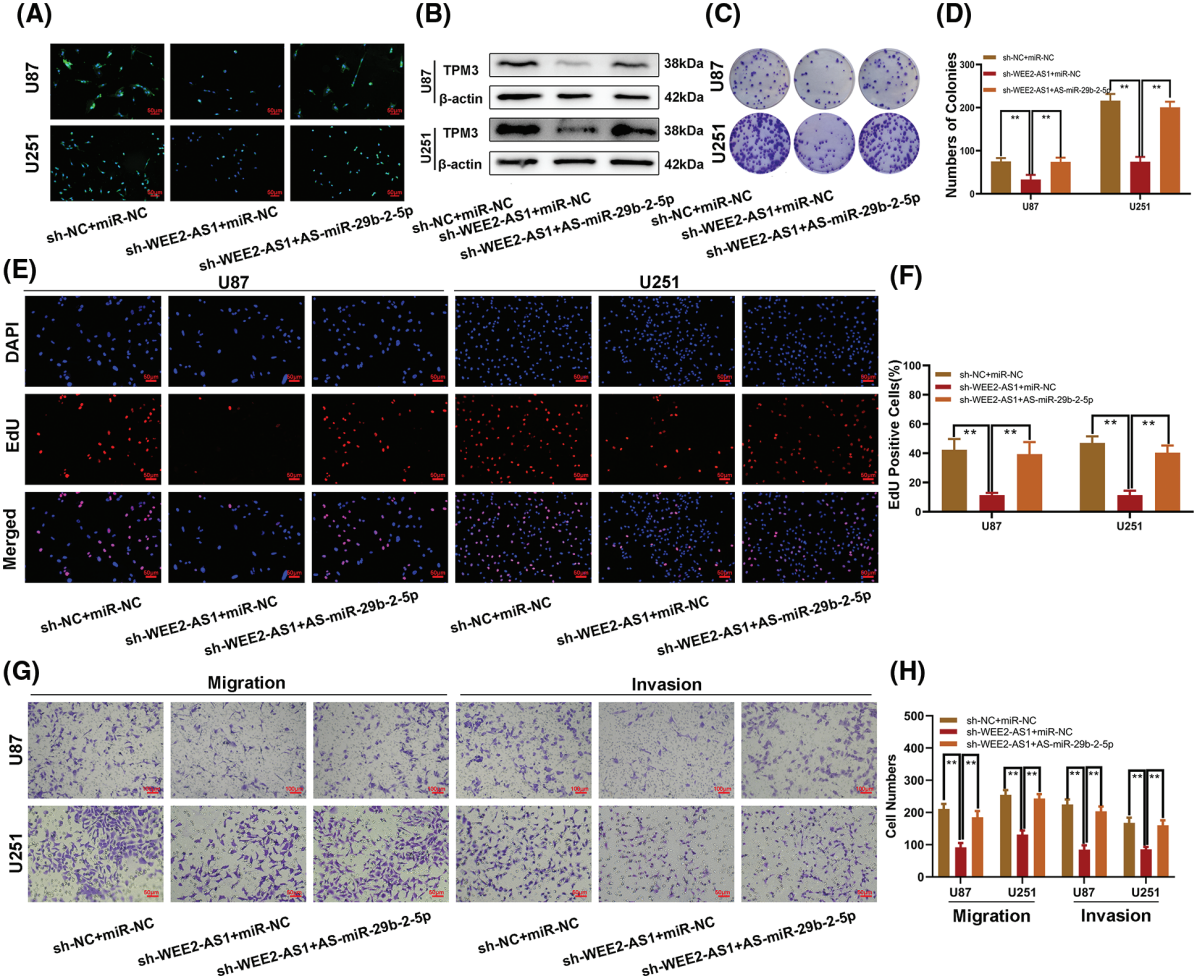
The effect of WEE2-AS1 on glioma is partially mediated by miR-29b-2-5p. (A, B) The relative expression of TPM3 in U87 and U251 cells transfected with miR-29b-2-5p inhibitor, sh-WEE2-AS1 or the corresponding controls, as measured by immunofluorescence and western blot. (C, D) The proliferation of U87 and U251 cells transfected with miR-29b-2-5p inhibitor, sh-WEE2-AS1 or the corresponding controls, as measured by clone formation assays. (E, F) The proliferation of U87 and U251 cells transfected with miR-29b-2-5p inhibitor, sh-WEE2-AS1 or the corresponding controls, as measured by EdU assays. (G, H) The migration and invasion of U87 and U251 cells transfected with miR-29b-2-5p inhibitor, sh-WEE2-AS1 or the corresponding controls, as measured by Transwell assays. Comparison was analyzed by one-way ANOVA for (D), (F) and (H). Data are expressed as the mean ± SD, **p < 0.01 from three independent experiments.

To confirm the functions of miR-29b-2-5p and WEE2-AS1 in glioma progression. sh-WEE2-AS1, three cell models were created using sh-WEE2-AS1, miR-29b-2-5p inhibitors, and a negative control. Immunofluorescence (Fig. 5A) and western blotting (Fig. 5B) demonstrated that downregulation of WEE2-AS1 inhibited the expression of TPM3 and that this effect could be restored by the miR-29b-2-5p inhibitor. Colony formation assays (Figs. 5C and 5D) and EdU (Figs. 5E and 5F) assays showed that cell proliferation was suppressed with sh-WEE2-AS1 transfection, while proliferation was rescued by miR-29b-2-5p downregulation. The Transwell assay yielded similar results, with the miR-29b-2-5p inhibitor reversing the impact of sh-WEE2-AS1 on migration and invasion in glioma cell lines (Fig. 5G and 5H). Taken together, our data demonstrate that WEE2-AS1 modulates the growth, migration, and invasion of glioma cell lines by sponging miR-29b-2-5p.

### TPM3 exerts its influential functions involving the proliferation, migration, and invasion of glioma cell lines via the WEE2-AS1/miR-29b-2-5p Axis

**Figure 6:**
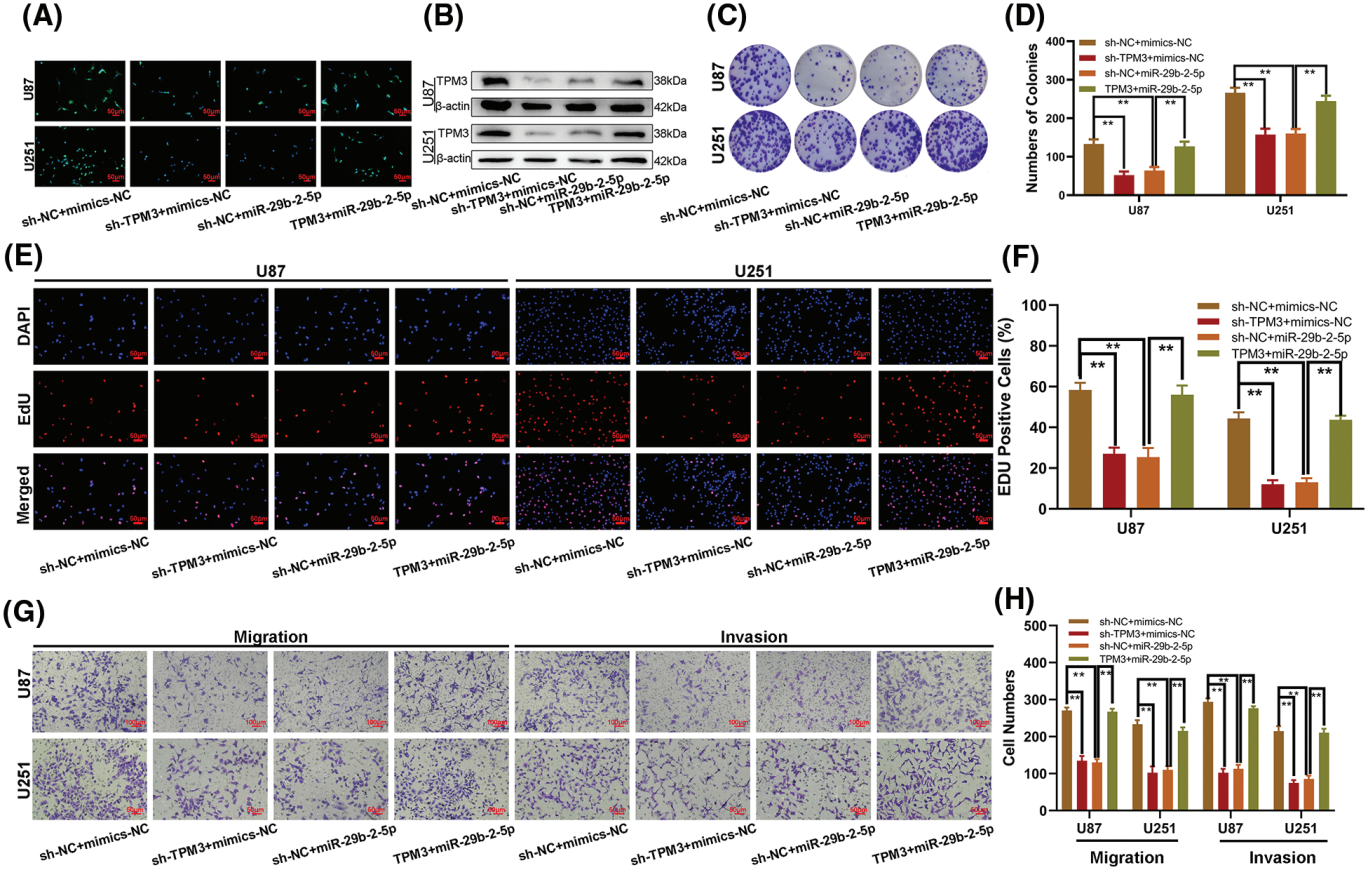
TPM3 promotes glioma proliferation, migration and invasion *in vitro*. (A, B) The relative expression of TPM3 in U87 and U251 cells transfected with sh-TPM3, miR-29b-2-5p mimics or TPM3 together with miR-29b-2-5p mimics, as measured by immunofluorescence and western blot. (C, D) The proliferation of U87 and U251 cells transfected with sh-TPM3, miR-29b-2-5p mimics or TPM3 together with miR-29b-2-5p mimics, as measured by clone formation assays. (E, F) The proliferation of U87 and U251 cells transfected with sh-TPM3, miR-29b-2-5p mimics or TPM3 together with miR-29b-2-5p mimics, as measured by EdU assays. (G, H) The migration and invasion of U87 and U251 cells transfected with sh-TPM3, miR-29b-2-5p mimics or TPM3 together with miR-29b-2-5p mimics, as measured by Transwell assays. One-way ANOVA and *post hoc* test for (D), (F), and (H). Data are expressed as the mean ± SD, **p < 0.01 from three independent experiments.

To explore whether TPM3 is the proper working target of the WEE2-AS1/miR-29b-2-5p axis in regulating glioma cell behaviors, four cell types were transfected with sh-TPM3, miR-29b-2-5p mimics, a TPM3 expression plasmid, and a matched negative control. Immunofluorescence (Fig. 6A) and western blot assays (Fig. 6B) indicated that sh-TPM3 decreased the expression level of TPM3, and a similar effect was observed in the miR-29b-2-5p-mimic groups, while the effect was counteracted by the TPM3 expression plasmid. Colony formation (Figs. 6C and 6D) and EdU assays (Figs. 6E and 6F) demonstrated the inhibitory effect of sh-TPM3 and miR-29b-2-5p-mimics on the proliferation of glioma cell lines, which could be reversed after treatment with the TPM3 expression plasmid. A similar result was obtained from the Transwell assay (Figs. 6G and 6H): cotransfection of TPM3 and miR-29b-2-5p mimics attenuated the inhibitory effect of miR-29b-2-5p mimic treatment on the migration and invasion of glioma cell lines after miR-29b-2-5p mimic treatment. From these findings, we deduced that TPM3 promoted the proliferative, migratory, and invasive functions of glioma cells *via* the WEE2-AS1/miR-29b-2-5p axis.

### Depletion of lncRNA WEE2-AS1 induced the suppressive effect of tumor growth in vivo

**Figure 7:**
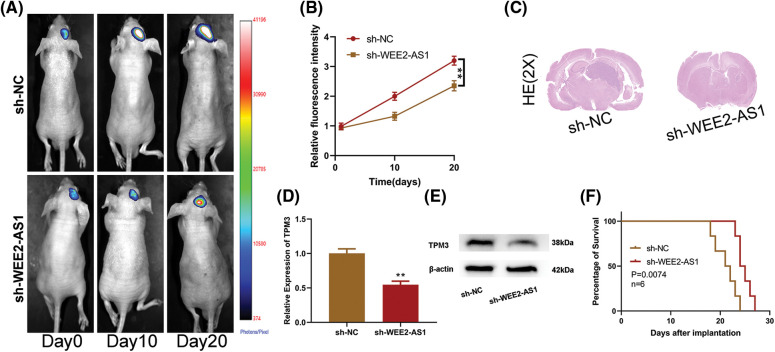
WEE2-AS1 promotes glioma growth *in vivo*. (A) *In vivo* imaging of intracranial tumors *in situ* in nude mice was acquired at days 1, 10 and 20 after implantation. (B) Relative fluorescence intensities were used to indirectly illustrate sh-WEE2-AS1 and sh-NC groups of tumor volumes in the brains of nude mice by Tannon Images software. (C) Hematoxylin-eosin staining of the maximum diameter of tumors between the sh-WEE2-AS1 and sh-NC groups. (D) The relative expression of TPM3 in nude mice implanted with sh-NC and sh-WEE2-AS1, as measured by qRT–PCR. (E) The relative expression of TPM3 in nude mice implanted with sh-NC and sh-TPM3, as measured by western blot. (F) Overall survival was compared between the sh-WEE2-AS1 and sh-NC groups by Kaplan–Meier survival curves. Two-way ANOVA and *post hoc* test for (B). Comparison was analyzed by *t*-test for (D). Data are expressed as the mean ± SD, **p < 0.01 from three independent experiments.

To assert the role WEE2-AS1 plays in tumor growth *in vivo*, we stereotactically injected U87-sh-WEE2-AS1 or U87-sh-NC expressing luciferase into the right frontal lobe of nude mice to generate *in situ* xenografts. The results implied that sh-WEE2-AS1 suppressed tumor growth in comparison to that observed in the sh-NC group (Fig. 7A). To indirectly depict tumor volume, the Tanon Image software program was utilized to estimate the relative fluorescence intensity in the brains of nude mice. The tumor fluorescence intensity in the sh-WEE2-AS1 group was substantially lower than in the sh-NC group. (Fig. 7B). Consistent with these findings, hematoxylin-eosin (HE) staining (Fig. 7C) of the maximum diameter of orthotopic graft tumors showed a similar effect. TPM3 was downregulated in the sh-WEE2-AS1 group relative to the NC group, as shown by qRT–PCR (Fig. 7D) and western blot (Fig. 7E). Furthermore, we noticed that mice in the sh-WEE2-AS1 group had a better chance of survival than mice in the NC group (Fig. 7F). In conclusion, our data indicates that inhibiting WEE2-AS1 suppresses glioma growth *in vivo*.

## Discussion

Historically, approximately 95% of the human genome’s nonprotein coding region was considered useless [20]. However, emerging evidence indicates that lncRNAs play critical roles in the establishment and evolution of a variety of malignancies [21]. A series of lncRNAs are considered as potential molecular biomarkers or future therapeutic targets [22]. LINC00662 acts as an oncogene, promoting the development of glioma by blocking the miR-340-5p/STAT3 axis. Meanwhile, STAT3 boosted LINC00662 transcription, which indicates that LINC00662 may be exploited as a molecular marker and therapeutic target for gliomas [23]. It has been reported H19 expression increases glioblastoma cell invasion, angiogenesis, stem cell production, and tumorigenesis that increasing H19 expression may promote glioblastoma cell invasion, angiogenesis, stem cell production, and tumorigenesis, and that H19 expression will become a potential target for glioblastoma therapy [24]. In an attempt to elucidate the mechanism behind the incidence and progression of glioma, lncRNAs have attracted great interest. To date, the molecular mechanism of the lncRNA/miRNA/mRNA network has been extensively studied in a variety of tumor types [25]. For instance, lncRNAH19 was demonstrated to upregulate VASH2 expression by inhibiting microRNA-29a and inducing angiogenesis [26]. LncRNA WEE2-AS1 was shown to be upregulated in triple-negative breast cancer and to act as an oncogene in the miR-32-5p/TOB1 axis [27]. LncRNAs are crucial in glioma genesis and progression.

Long noncoding RNAs may impact the growth of glioma cells through chromatin alteration [28]. NEAT1 may interact with EZH2 and mediate H3K27 trimethylation in their promoters, and NEAT1 was essential for glioma cell proliferation and invasion, since it increased β-catenin nuclear transport while decreasing ICAT, GSK3B, and Axin2 expression [29]. Furthermore, many lncRNAs have been shown to encode functional micropeptides based on small-ORFs [30]. The phosphorylation of the translation initiation factor eIF4E by TNF and mammalian ste20-like kinase activates a stress and tumor necrosis factor (TNF)-activated ORF micropeptide (STORM) derived from Linc00689 [31]. Moreover, exosome-transmitted lncRNAs can play roles in intercellular communication and regulate the tumor microenvironment [32,33]. LINC00470 in GBM- exosome can bind to miR-580-3p in glioma cells and stimulate the PI3K/AKT/mTOR pathway, impeding autophagy and promoting glioma cell proliferation [34].

We established that WEE2-AS1 expression is increased in glioma cell lines and tissues in the current investigation, which corroborates the information in the GEPIA database. WEE2-AS1 knockdown repressed the proliferation, migration and invasion of U87 and U251 cells, but upregulation of WEE2-AS1 had the reverse impact. To determine the real involvement of WEE2-AS1 in glioma tumorigenesis, we conducted FISH assays, which confirmed the subcellular localization of WEE2-AS1 in glioma cell lines, implying that WEE2-AS1 may participate in posttranscriptional regulation. MiR-29b-2-5p was identified as a potential gene using a luciferase reporter experiment following bioinformatics analysis. Likewise, miR-29b-2-5p has been reported to inhibit cancer growth in pancreatic ductal adenocarcinoma by influencing Cbl-b [14]. In glioma tissues and cell lines, we confirmed that miR-29b-2-5p expression was decreased. Bioinformatics technology analysis was used to confirm the downstream gene TPM3. The mammalian genome contains four independent tropomyosin genes: tropomyosin 1 (TPM1), tropomyosin 2 (TPM2), tropomyosin 3 (TPM3), tropomyosin 4 (TPM4), and more than 40 different tropomyosin isomers are produced by alternative splicing [35]. Some high molecular weight TPMs (TPM1, TPM2 and TPM3) play a key role in stabilizing actin filaments and regulating cell morphology and division [36]. In recent years, TPM3 has been reported in many tumor diseases, including esophageal cancer, breast cancer and pancreatic cancer, but rarely reported in glioma [18,37,38]. A luciferase reporter assay revealed that WEE2-AS1 mediates the downstream gene TPM3 by sponging miR-29b-2-5p. Downregulation of miR-29b-2-5p rescued the TPM3 downregulation caused by knockdown of WEE2-AS1 and the suppression of cell proliferation, migration and invasion in glioma.

In summary, high expression of WEE2-AS1 was found in glioma cell lines and tissues, and downregulation of WEE2-AS1 induced the suppression of glioma cell proliferation, migration and invasion. WEE2-AS1 functions as a critical oncogenic lncRNA through its regulatory role in the WEE2-AS1/miR-29b-2-5p/TPM3 axis. Studies of the effect of noncoding RNAs on gliomas appear to be in the early stage of discovery. However, the role of WEE2-AS1 overexpression and TPM3 knockdown or overexpression *in vivo* remains to be further studied. Additionally, whether WEE2-AS1 can be exploited as a molecular marker or therapeutic target in GBM requires further exploration in future investigations. Our findings may provide novel insights that can guide the development of effective molecular therapies against glioma and facilitate a better understanding of glioma pathogenesis.
